# Prognostic and Predictive Role of Circulating Tumor DNA in Locally Advanced Rectal Cancer Managed With Total Neoadjuvant Therapy: A Systematic Review

**DOI:** 10.1002/hsr2.72905

**Published:** 2026-07-28

**Authors:** Faizan Bashir, Qurat‐ul‐Aine Bhat, Jayden Allegakoen, Dorsa Shekouh, Sajida Zaiter

**Affiliations:** ^1^ School of Medicine Shiraz University of Medical Sciences Shiraz Iran; ^2^ Department of Biomedical Engineering Johns Hopkins University Baltimore Maryland USA

**Keywords:** circulating tumor DNA, ctDNA, LARC, rectal cancer, TNT, total neoadjuvant therapy

## Abstract

**Background and Aims:**

Circulating tumor DNA (ctDNA) has emerged as a promising biomarker of tumor burden and minimal residual disease. As total neoadjuvant therapy (TNT) increasingly supports organ‐preserving strategies in locally advanced rectal cancer (LARC), reliable markers of treatment response are needed. This review evaluates the prognostic and predictive value of ctDNA in this setting.

**Methods:**

Following PRISMA guidelines, we systematically reviewed studies across PubMed/MEDLINE, Web of Science, Cochrane and Google Scholar evaluating ctDNA during or after TNT in LARC reporting treatment‐response or survival outcomes.

**Results:**

Seven studies met inclusion criteria. Baseline ctDNA detection was near‐universal (83–100%) but lacked discriminatory value. ctDNA declined progressively during TNT, with post‐TNT negativity correlating with clinical or pathological complete response (*p* = 0.007–0.038). Persistent positivity predicted residual disease and inferior survival (DFS HR 4.03; OS HR 23.0), with presurgical ctDNA in GEMCAD showing preferential association with hepatic recurrence suggesting a marker of systemic rather than locoregional disease. Post‐TNT ctDNA demonstrated high specificity but low sensitivity (23%–45%) for residual disease, and preceded clinical recurrence detection by weeks to months.

**Conclusions:**

ctDNA clearance at the end of TNT may represent a biologically meaningful marker of treatment response and recurrence risk. However, current data derive from small, heterogeneous cohorts with variable assay platforms and sampling schedules, and ctDNA's limited sensitivity precludes its use as a standalone tool for non‐operative management selection.

**Abbreviations**
Bev: bevacizumab
CAPOX: capecitabine plus oxaliplatin
cCR: clinical complete response
CRT: chemoradiotherapy
DFS: disease‐free survival
FOLFOX: 5‐fluorouracil, leucovorin, oxaliplatin
LCCRT: long‐course chemoradiotherapy
mFOLFOX6: modified FOLFOX regimen (6‐cycle schedule)
MRD: minimal residual disease
NOM: non‐operative management
OS: overall survival
pCR: pathological complete response
SCRT: short‐course radiotherapy
TNT: total neoadjuvant therapy

## 1 Introduction

Colorectal cancer remains the second leading cause of cancer‐related mortality worldwide, accounting for approximately 10% of incident cases and 9% of deaths globally, with projections estimating 3.2 million new cases and 1.6 million deaths annually by 2040. Locally advanced rectal cancer (LARC), defined as tumors extending beyond the muscularis propria (cT3–T4) or involving regional lymph nodes (stage II–III) represents a clinically distinct and therapeutically challenging subset, in which accurate response assessment and individualized treatment planning are paramount [1, 2, 3, 4].

Management of LARC relies on a multimodal approach combining chemotherapy, chemoradiotherapy, and surgery, though optimal sequencing of these components remains an area of active refinement [5]. The adoption of total neoadjuvant therapy (TNT), in which systemic chemotherapy and chemoradiotherapy are delivered prior to surgery, has reshaped treatment strategies. Compared with conventional sequencing, TNT improves treatment adherence, increases rates of pathological complete response (pCR) and disease‐free survival (DFS), and supports the selective use of nonoperative approaches [6, 7]. Despite these advances, treatment response remains heterogeneous; some patients derive limited benefit or experience early progression, highlighting the need for more reliable predictors of therapeutic response [8]. Alongside molecular biomarkers, imaging‐based tools have also been explored. In particular, PET/CT‐derived parameters such as metabolic tumor volume (MTV) and total lesion glycolysis (TLG) have shown associations with treatment response, with lower values observed in patients achieving complete response [9].

Circulating tumor DNA (ctDNA), consisting of short fragments of tumor‐derived DNA released into the bloodstream, has emerged as a minimally invasive biomarker for disease monitoring and risk stratification [10]. Its clinical utility has been demonstrated across gastrointestinal malignancies. For instance, in pancreatic ductal adenocarcinoma, detectable ctDNA following chemotherapy has been associated with subsequent radiologic progression in a substantial proportion of patients, compared with lower rates among ctDNA‐negative individuals [11]. Similarly, in colorectal cancer, ctDNA positivity is strongly associated with recurrence risk, supporting its role in refining adjuvant treatment decisions [12].

In the context of TNT‐treated LARC, ctDNA may offer additional insight into treatment response [13] and residual disease across multiple timepoints, including baseline, during therapy, and after treatment completion. However, current evidence remains fragmented, reflecting variability in sampling strategies, detection platforms, and outcome definitions [14]. Furthermore, the populations studied are heterogeneous, encompassing patients who proceed to surgical resection as well as those managed non‐operatively following clinical response.

This systematic review therefore evaluates the prognostic and predictive value of ctDNA dynamics during TNT in patients with LARC undergoing either surgical resection or non‐operative management.

## 2 Methods

### 2.1 Protocol Registration

This systematic review was conducted in accordance with the Preferred Reporting Items for Systematic Reviews and Meta‐Analyses (PRISMA) guidelines [15], and was prospectively registered in PROSPERO under the registration number CRD420251164506.

### 2.2 Search Strategy

A comprehensive search of the literature was performed in MEDLINE/PubMed, Web of Science, Cochrane Library and Google Scholar from database inception through October 2025. This search strategy combined Medical Subject Headings (MeSH) and terms related to rectal cancer, LARC, total neoadjuvant therapy, and circulating tumor DNA. We also screened the reference lists of relevant articles to capture any potentially missed studies. Our search strategy did not filter for language, date or type of study. The complete search strategy employed for each database is displayed in Table 1.

**Table 1:** Database‐specific search strings and the number of records retrieved until October 2025.

Database	Search strategy	Records found
MEDLINE/PubMed	((“circulating tumor DNA”[Title/Abstract] OR “circulating tumor DNA”[Title/Abstract] OR ctDNA[Title/Abstract] OR “cell free DNA”[Title/Abstract] OR cfDNA[Title/Abstract]) AND (“total neoadjuvant”[Title/Abstract] OR TNT[Title/Abstract] OR “neoadjuvant chemoradiotherapy”[Title/Abstract] OR “neoadjuvant therapy”[Title/Abstract]) AND (“rectal cancer”[Title/Abstract] OR “rectal carcinoma”[Title/Abstract] OR “rectal neoplasms”[MeSH] OR “locally advanced rectal cancer”[Title/Abstract] OR LARC[Title/Abstract]))	57
Web of Science	TS = ((“circulating tumor DNA” OR “circulating tumor DNA” OR ctDNA OR “cell free DNA” OR cfDNA) AND (“total neoadjuvant” OR TNT OR “neoadjuvant chemoradiotherapy” OR “neoadjuvant therapy”) AND (“rectal cancer” OR “rectal carcinoma” OR “rectal neoplasms” OR “locally advanced rectal cancer” OR LARC))	83
Cochrane	(locally advanced rectal cancer):ti, ab, kw AND (ctDNA):ti, ab, kw OR (circulating tumor DNA):ti, ab, kw AND (total neoadjuvant therapy):ti, ab, kw	53
Google Scholar	allintitle: circulating tumor dna locally advanced rectal cancer total neoadjuvant therapy	7

### 2.3 Study Selection

During this step, two reviewers (S.Z. and Q.A.B) independently screened all titles and abstracts for relevance. Full‐text articles were retrieved for studies that appeared to meet inclusion criteria or for which eligibility was uncertain. Disagreements were resolved through discussion and consensus, with input from a third reviewer (F.B) when necessary.

### 2.4 Eligibility Criteria

Studies were included if they met all of the following criteria:

1. Enrolled patients with locally advanced rectal cancer (cT3–T4 or node‐positive, M0) treated with total neoadjuvant therapy (TNT), defined as systemic chemotherapy and chemoradiotherapy delivered entirely before surgery or non‐operative management, including patients managed with a watch‐and‐wait approach following clinical response.
2. Reported data on circulating tumor DNA (ctDNA) measured at one or more defined timepoints during or after TNT using validated tumor‐informed or tumor‐naïve assays.
3. Evaluated at least one of the following outcomes:
  - Predictive: clinical or pathologic complete response (cCR or pCR), response to TNT, or organ preservation/non‐operative management (NOM).
  - Prognostic: recurrence, disease‐free survival (DFS), progression‐free survival (PFS), or overall survival (OS).
4. Published in English, non‐english texts were excluded after screening.

Exclusion criteria included studies that:

- Involved non‐rectal primary tumors or metastatic disease at baseline.
- Reported ctDNA data outside the TNT setting (e.g., adjuvant or metastatic contexts).
- Were review articles, isolated case reports, or laboratory‐only studies lacking clinical outcomes.
- Studies published in languages other than English.

### 2.5 Data Extraction and Management

Two reviewers (Q.A.B. and F.B.) independently extracted all data using a standardized form designed a priori. Extracted variables included study design, country, year, patient characteristics, TNT regimen details, ctDNA assay type and sampling timepoints, criteria for ctDNA positivity or clearance, and clinical outcomes (cCR, pCR, NOM, recurrence, DFS, PFS, OS). Statistical measures such as hazard ratios (HRs), effect sizes, and confidence intervals (CIs) were recorded when provided. Discrepancies were resolved by consensus.

### 2.6 Quality Assessment

The methodological quality of each included study were appraised using the Joanna Briggs Institute (JBI) Critical Appraisal Checklists according to the study design (https://jbi.global/critical-appraisal-tools). Quality assessment of each study was done by two reviewers (D.S and F.B). All disagreements during this phase were resolved through consensus.

### 2.7 Data Synthesis

Findings were synthesized narratively and organized into three domains: (1) predictive value of ctDNA for treatment response and organ preservation; (2) prognostic value for recurrence and survival; and (3) assay characteristics and optimal timing of ctDNA assessment. When possible, the direction and strength of associations between ctDNA status and clinical endpoints were summarized descriptively, and studies reporting comparable outcomes were grouped for thematic comparison. A meta‐analysis was not conducted due to substantial heterogeneity across studies in TNT regimens, ctDNA assays, sampling schedules, and outcome definitions.

### 2.8 Reporting Standards

All steps of the review process, from literature search to data synthesis, adhered to PRISMA 2020 standards.

### 2.9 Software Used

Search results from all the databases were imported to a referencing manager (Zotero). Following that all phases of study selection, screening and duplicate removal were performed manually, automation tools were not employed.

## 3 Results

### 3.1 Study Overview

**Figure 1:**
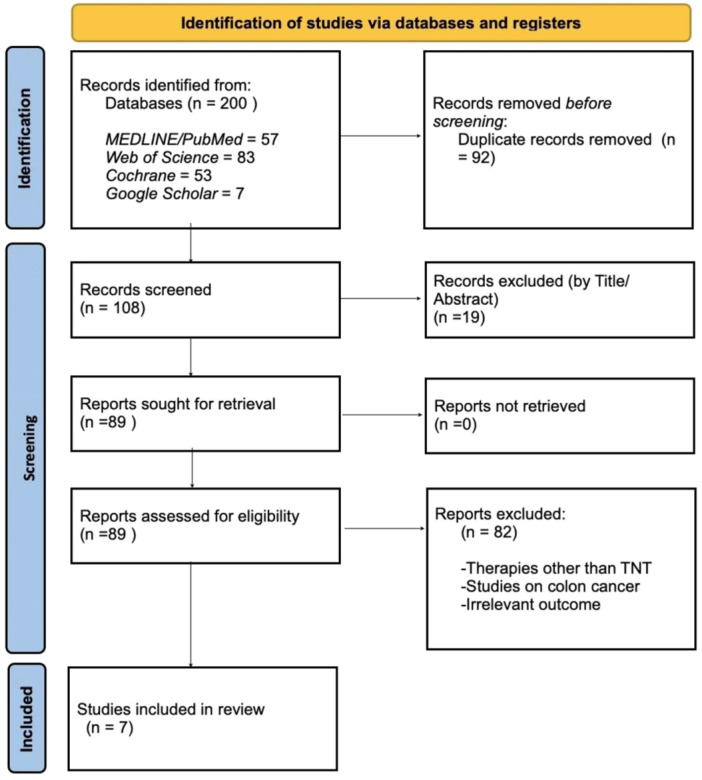
PRISMA flow diagram documenting the identification, screening, eligibility assessment, and final inclusion.

As detailed in the PRISMA flow diagram **(**Figure 1), a total of 200 records were identified through MEDLINE/PubMed (*n* = 57), Web of Science (*n* = 83), Cochrane (*n* = 53) and Google Scholar (*n* = 7). Following duplicate record removal and title/abstract screening, 89 unique records were thoroughly evaluated based on the eligibility criteria. Finally seven studies met the inclusion criteria for this review. These studies explored the role of ctDNA dynamics in the setting of TNT for locally advanced rectal cancer (LARC) with sample size ranging from 20 to 64 patients with analyzable ctDNA, including the biomarker substudy of the 180‐patient GEMCAD RCT [16, 17]. Five were prospective studies (including multicenter phase II trials and biomarker companion analyses) and two were retrospective analyses. Follow‐up durations varied and were limited in several biomarker cohorts.

The included studies encompassed heterogeneous post‐total neoadjuvant therapy (TNT) management strategies, ranging from definitive surgical cohorts to those incorporating organ‐preserving approaches. While some cohorts consisted of patients undergoing planned surgical resection following TNT to enable pathologic assessment [17] (e.g., Vidal et al. [GEMCAD 1402]), others explored the role of ctDNA in guiding organ preservation and potential watch‐and‐wait strategies following clinical response assessment [18] (e.g., Kim et al.). Several studies incorporated both approaches within the same cohort, where surgical resection was reserved for patients with an incomplete clinical response or local regrowth during surveillance [19, 20, 21] (e.g., Akiyoshi et al. [NOMINATE] and the ENSEMBLE studies). In contrast, other studies, such as Alden et al. and Felder et al., emphasized longitudinal ctDNA monitoring across multiple treatment timepoints, reflecting variability in post‐TNT management strategies [16, 22]. The therapeutic regimens varied across studies but uniformly followed a TNT framework, delivering both systemic chemotherapy and radiotherapy prior to surgical resection or confirmed non‐operative management. Approaches included short‐course radiotherapy (SCRT) followed by consolidation chemotherapy and long‐course chemoradiotherapy (LCCRT) administered in combination with systemic regimens such as FOLFOX or CAPOX. Certain protocols incorporated induction chemotherapy before chemoradiation, while others integrated adaptive radiotherapy boosts or structured organ‐preservation strategies within a selective watch‐and‐wait pathway [16, 17, 19, 20, 21, 22]. Most investigations used the tumor‐informed Signatera assay to monitor ctDNA at serial points, whereas one study (Vidal et al.) [17] employed an ultrasensitive plasma assay integrating genomic and epigenomic signatures (LUNAR‐1). Key study characteristics and further details of each study are provided in Table 2.

**Table 2:** Overview of the included studies reporting on ctDNA in the context of total neoadjuvant therapy (TNT). For each study, the table summarizes the clinical design, patient population (*N*), therapeutic protocol, ctDNA platform used, timing of serial assessments, and the principal oncologic outcomes measured.

Study	Design	*N*	TNT regimen	ctDNA assay	Key timepoints	Main outcomes measured
Felder et al., 2025	Prospective single arm clinical trial	20	CRT 50.4 Gy + FOLFOX	Signatera	Baseline, mid‐ CRT, post TNT	Clinical response, organ preservation, residual disease
Kim et al., 2025	Retrospective case series	28	SCRT/LCCRT + FOLFOX or CAPOX	Signatera	Post‐TNT, surveillance	MRD, cCR, NOM outcomes, recurrence
Vidal et al., 2021(GEMCAD 1402)	Cohort (Nested in RCT)	62	mFOLFOX6 ± aflibercept→ CRT	LUNAR‐1	Baseline, pre‐surgery	pCR, DFS, OS, recurrence
Kagawa et al., 2024 (ENSEMBLE‐1)	Prospective, single‐arm, phase II interventional clinical trial	30	SCRT + CAPOX	Signatera	Baseline, mid‐ TNT, post‐TNT	cCR, pCR, NOM
Alden et al., 2024	Retrospective	44	Short/long course RT + CAPOX/FOLFOX	Signatera	Pre/post‐TNT	Residual disease, MRI/endoscopy correlation
Akiyoshi et al., 2025 (NOMINATE)	Prospective, multicenter	64	CRT → CAPOX or Induction CAPOX+ Bev→CRT → CAPOX	Signatera	Baseline, interim, restaging, post‐TNT	cCR, pCR, NOM, local regrowth, distant metastases, DFS
Watanabe et al., 2025 (ENSEMBLE‐2)	Prospective, single‐arm, phase II interventional clinical trial	28	LCCRT + CAPOX	Signatera	Baseline, post‐LCCRT, post‐TNT, post‐op	cCR, pCR, NOM eligibility

### 3.2 ctDNA Kinetics During TNT

**Figure 2:**
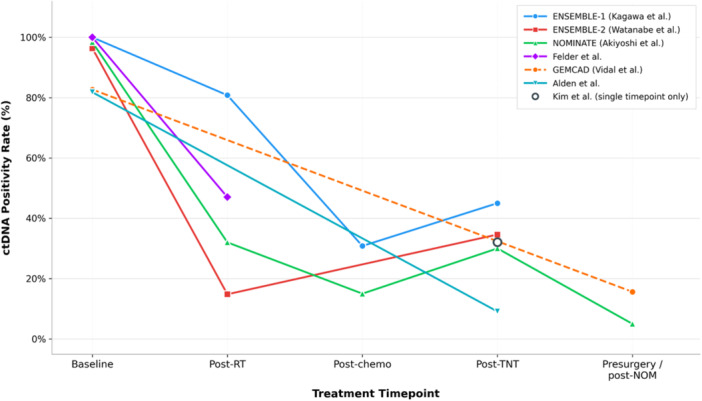
Serial ctDNA positivity rates (%) at defined assessment timepoints during total neoadjuvant therapy (TNT). Dots represent measured observations; connecting lines are shown for visual clarity only and do not imply continuous or uniform data collection. Timepoint definitions and sampling intervals varied across studies. The dashed line (GEMCAD, Vidal et al.) spans baseline to presurgery only, Kim et al. reported a single post‐TNT timepoint (open circle; 9/28 patients, 32.1%) and is displayed without a connecting line.

Across the prospective cohorts, almost all patients had detectable ctDNA before treatment. Baseline ctDNA detection was high across studies, ranging from 83% in GEMCAD [17] to 100% in Felder [16] and ENSEMBLE‐1, with ENSEMBLE‐2 reporting 96.4% and NOMINATE reporting 98.4% [19, 20, 21]. A sharp decline in ctDNA levels was consistently observed during therapy. In the ENSEMBLE‐1 trial [20], ctDNA positivity dropped from 100% at baseline to 81% after SCRT, 31% after four cycles of CAPOX, and 45% at completion of TNT. The ENSEMBLE‐2 study reported a comparable pattern, falling from 96.4% at baseline to 14.8% after LCCRT and 34.6% after TNT [21] (Figure 2).

The NOMINATE biomarker study also demonstrated sequential clearance, from 98.4% detection at baseline to 32% at the first interim evaluation, 15% at the second, and only 5% after TNT or during non‐operative management [19]. Likewise, Felder et al. noted that over half of their patients (53%) achieved ctDNA negativity midway through chemoradiation at 27 Gy; notably, every one of these early converters maintained rectal preservation at follow‐up. Notably, among patients who remained ctDNA‐positive at the mid‐CRT timepoint, 57% subsequently underwent formal total mesorectal excision with adenocarcinoma confirmed on pathology [16].

A structured synthesis of the clinical roles of ctDNA across the TNT continuum is presented in Table 3.

**Table 3:** Summary of how ctDNA status at different timepoints during total neoadjuvant therapy relates to treatment response, residual disease, and recurrence risk in locally advanced rectal cancer.

Domain	Timing of assessment	Findings reported across included studies	Clinical relevance/Purpose
Sampling and detection	Baseline; during TNT; post‐TNT/presurgery	Baseline ctDNA detection ranged from 83% to 100% across studies. Most cohorts used tumor‐informed assays (e.g., Signatera), while one substudy (GEMCAD) used a plasma‐based assay (LUNAR‐1).	Establishes measurable tumor‐derived DNA at diagnosis and enables serial molecular monitoring during treatment.
Treatment response assessment	Mid‐treatment; post‐TNT	ctDNA levels declined during TNT in prospective cohorts. ctDNA negativity at completion of TNT was associated with (cCR) and/or (pCR) in several studies. Persistent post‐TNT ctDNA detection was associated with residual disease or increased likelihood of requiring surgery after attempted non‐operative management.	Supports assessment of molecular response and may aid in identifying candidates for organ‐preserving strategies.
Prognostic significance	Presurgery; post‐TNT	Presurgical or post‐TNT ctDNA positivity was associated with higher recurrence risk and inferior survival in biomarker substudies (e.g., DFS HR ~ 4.0; OS HR up to ~23.0 in GEMCAD). Studies reporting diagnostic metrics demonstrated high specificity for residual disease, with variable sensitivity.	Enables risk stratification beyond radiologic and pathologic assessment alone.
Surveillance and recurrence monitoring	Post‐treatment follow‐up	Re‐emergent or persistent ctDNA detection was reported in patients who subsequently developed local regrowth or systemic recurrence in non‐operative management pathways.	May contribute to early identification of recurrence during follow‐up.
Biological basis	Continuous	ctDNA is derived from tumor cell apoptosis and necrosis. Its short half‐life (mins to hrs) allows near real‐time assessment of tumor dynamics.	Provides a dynamic molecular complement to imaging and pathologic evaluation.

### 3.3 Predictive Value for Response

Several studies linked ctDNA clearance during TNT to favorable local tumor response. In ENSEMBLE‐1, ctDNA negativity after SCRT and after four CAPOX cycles correlated with clinical complete response (both *p* = 0.007), while negativity at the end of TNT was significantly associated with pathologic complete response (pCR) (*p* = 0.029) [20]. The ENSEMBLE‐2 trial yielded nearly identical findings—post‐TNT ctDNA negativity predicted both combined clinical + near‐complete response (*p* = 0.028) and pCR (*p* = 0.038), whereas ctDNA drawn immediately after LCCRT did not show predictive value. It is also notable that in ENSEMBLE‐2, the association between post‐TNT ctDNA status and the decision to pursue NOM versus surgery trended toward but did not reach statistical significance (*p* = 0.063), indicating that ctDNA alone was not yet sufficient to drive the organ‐preservation decision in this cohort [21].

Within the NOMINATE study, patients achieving a clinical or near‐complete response who pursued non‐operative management exhibited complete ctDNA clearance from interim to post‐therapy assessments. Non‐responders (non‐cCR) demonstrated lower clearance rates (75% and 51% at mid‐ and post‐TNT, respectively) [19]. Felder et al. reached a similar conclusion: all patients who converted to ctDNA negativity midway through CRT maintained organ preservation, suggesting that early clearance could identify candidates for watch‐and‐wait strategies [16].

Retrospective data supported these findings, albeit more modestly. In a single‐institution series, Kim et al. [18] observed that two‐thirds of ctDNA‐positive patients after TNT required surgery for residual cancer compared with one‐fifth of ctDNA‐negative individuals (67% vs. 21%, *p* = 0.035). Furthermore, in the same cohort, ctDNA testing appeared to detect distant metastases before conventional imaging in two patients, with ctDNA turning positive weeks ahead of CT‐confirmed metastatic disease. In contrast, Alden et al. [22] evaluated post‐TNT patients and reported limited sensitivity of ctDNA testing for residual disease (23%) despite perfect specificity (100%), yielding a PPV of 100% and an NPV of 47%. The authors concluded that ctDNA negativity alone could not safely identify complete responders (those without local regrowth). This study also evaluated ctDNA against MRI tumor regression grade (mrTRG) and endoscopic response: ctDNA showed sensitivity of 16% and specificity of 96% for predicting poor tumor regression on MRI, with a PPV of 75% and NPV of 60%, and sensitivity of only 5% for predicting residual disease on endoscopy. Detailed ctDNA kinetics, timepoint positivity and their relation to response and prognosis across studies are shown in Table 4.

**Table 4:** Relationship between ctDNA status at key treatment timepoints and response or recurrence outcomes during total neoadjuvant therapy.

Study	Timepoint	ctDNA status	Outcome association
Felder et al.	Mid‐CRT	Negative	Early ctDNA clearance associated with organ preservation, cCR
Kim et al.	Post‐TNT	Positive	67% required surgery, MRD detected, ctDNA detected distant metastases before CT imaging in 2/3 patients with metastatic disease
Vidal et al.	Presurgery	Positive	HR recurrence 4.03; OS HR 23.0
ENSEMBLE‐1	After 4 cycles CAPOX	Negative	Predicted cCR (*p* = 0.007)
Alden et al.	Post‐TNT	Positive	Residual disease PPV 100%, sensitivity 23%
NOMINATE	Post‐TNT	Positive	Associated with shorter DFS (HR 6.7–10.2)
ENSEMBLE‐2	Post‐TNT	Negative	Associated with cCR, pCR, potential NOM eligibility

Collectively, these findings indicate that persistent ctDNA positivity during or after TNT reflects suboptimal tumor regression, whereas ctDNA clearance is closely associated with pathological or clinical complete response and may support decisions regarding organ preservation.

### 3.4 Prognostic Significance

Two prospective studies extended these observations to systemic outcomes. In the GEMCAD‐1402 ctDNA substudy, presurgery ctDNA positivity was observed in 15% of evaluable patients and was associated with a higher risk of systemic recurrence (DFS HR 4.03; *p* = 0.033) and worse overall survival (OS HR 23.0; *p* < 0.0001) among the substudy cohort (72 paired samples; 62 sequenced). This study also found that presurgery ctDNA positivity was detected in 75% of patients who subsequently developed liver metastases, compared with only 9.8% of those who never developed hepatic recurrence (*p* = 0.009), supporting the concept that presurgery ctDNA specifically reflects systemic minimal metastatic disease rather than local tumor burden — a distinction the authors termed “minimal metastatic disease” (MMD) to differentiate it from locoregional minimal residual disease [17]. Similarly, the NOMINATE companion analysis found that ctDNA detection at final restaging predicted residual pathologic disease with 100% specificity and positive predictive value, and was associated with shorter disease‐free survival at T3 (HR 6.7; *p* = 0.005) and at T4 (HR 10.2; *p* = 0.0005) [19].

Follow‐up surveillance data reinforced the prognostic role of ctDNA. Among patients managed non‐operatively in NOMINATE, five experienced local regrowth, and ctDNA reappeared in two of those cases. In one of these cases, ctDNA became detectable 84 days before clinical confirmation of local regrowth, and was accompanied by synchronous lung metastasis at the time of salvage surgery. Additionally, ctDNA detected all three cases of distant metastasis with 100% sensitivity in patients with available testing, further supporting its role as an early systemic recurrence signal [19]. In the Moffitt cohort, patients who remained ctDNA‐negative continued to maintain organ preservation, although long‐term outcomes are still maturing [16].

### 3.5 Assay and Timing Considerations

Most studies [16, 18, 19, 20, 21, 22] used tumor‐informed assays (Signatera) that demonstrated high baseline detection in several cohorts (e.g., Felder and ENSEMBLE‐1 observed 100% baseline detection; ENSEMBLE‐2 96.4%; NOMINATE 98.4%). The GEMCAD substudy used a plasma‐only genomic/epigenomic assay (LUNAR‐1) and detected baseline ctDNA in 83% of patients. It should be noted that in GEMCAD, 31% of samples failed quality control analysis, largely due to insufficient plasma volume, which may have reduced assay sensitivity and should be considered when interpreting the 15% post‐TNT ctDNA detection rate in that cohort [17]. The LUNAR‐1 assay was prognostic for DFS and OS in GEMCAD but did not correlate with pCR; across studies, post‐TNT sampling provided the most consistent correlation with pCR and long‐term outcomes.

### 3.6 Summary of Evidence

Across these reports, ctDNA negativity after TNT was associated with favorable local and, in selected cohorts, systemic outcomes. Serial ctDNA monitoring captured treatment response in real time, identified patients likely to achieve complete remission, and signaled recurrence risk when re‐emergent. Persistent or recurrent ctDNA positivity was associated with residual disease and, in prospective biomarker cohorts, inferior survival. Although consistent use of tumor‐informed assays strengthens the biological signal, heterogeneity in TNT regimens, timing of sampling, and response definitions precluded quantitative synthesis. Nevertheless, the collective evidence provides a coherent rationale for integrating ctDNA assessment into modern TNT algorithms and post‐treatment surveillance in LARC.

### 3.7 Quality of Studies

The methodological quality of the included studies was assessed using the appropriate JBI critical appraisal tools based on study design. Overall, all full‐text studies indicated high methodological quality. The single case series study [18], completed all ten JBI criteria (10/10), demonstrating a high‐quality rating. Among the three cohort studies [17, 19, 22], all completed most of the JBI criteria, Akiyoshi et al. (NOMINATE) met 11/11 criteria, Alden et al. satisfied 8/11 criteria, and the GEMCAD substudy met 9/11 criteria. Two studies [17, 22] had unclear confounding, possibly indicating bias due to confounding factors, whereas the other items were comprehensively addressed. However, there were some minor limitations; both were rated as high quality overall. Three records [16, 20, 21] were assessed as unclear risk, because detailed methodological information, particularly details about patient selection, exposure measurement, follow‐up adequacy, and strategies for managing confounding could not be verified.

## 4 Discussion

In this systematic review, ctDNA emerged as a biologically coherent marker of treatment response in LARC treated with TNT. Despite heterogeneity in protocols, sampling schedules, and response definitions, the findings were strikingly consistent: patients who achieved ctDNA clearance after TNT had more favorable local and systemic outcomes, while those with persistent or re‐emergent positivity faced higher rates of residual disease, metastatic progression, and shorter survival.

However, ctDNA negativity should not be interpreted as definitive evidence of complete disease eradication. Across well‐characterized rectal cancer cohorts, a meaningful proportion of ctDNA‐negative patients have nonetheless experienced disease relapse — a finding that reflects both the biological complexity of minimal residual disease and the inherent sensitivity constraints of current assay platforms [18, 23, 24]. This underscores the importance of contextualizing ctDNA results within the broader clinical picture, particularly when informing decisions around treatment de‐escalation or non‐operative management.

At the biological level, ctDNA comprises tumor‐derived DNA fragments released through apoptosis, necrosis, and active secretion. Its concentration correlates with tumor burden, proliferation, vascularity, and metastatic potential; for example, liver metastases are associated with higher ctDNA shedding [25, 26]. Because circulating cell‐free DNA includes a background from normal cells, tumor‐specific alterations such as somatic mutations or methylation signatures are required to distinguish ctDNA from non‐tumor cfDNA [27]. With a half‐life measured in minutes to hours, ctDNA provides a near–real‐time measure of tumor dynamics [10], offering the potential to detect treatment response and emerging resistance earlier than radiologic assessment.

Across the seven included studies, several consistent themes emerged. Baseline ctDNA positivity was almost universal, underscoring the feasibility of molecular monitoring in LARC. All prospective trials showed substantial declines during TNT ENSEMBLE‐1, ENSEMBLE‐2, NOMINATE, and Felder et al. each demonstrated early reductions that paralleled clinical response while persistent ctDNA [16, 19, 20, 21], whether detected post‐TNT in Kim et al. [18] or presurgery in GEMCAD [17], was uniformly associated with residual disease, early recurrence, or inferior survival. A particularly noteworthy finding from NOMINATE was the re‐emergence of ctDNA positivity between the second interim evaluation and final restaging in eight patients, potentially reflecting the emergence of tumor clones resistant to radiotherapy or systemic chemotherapy—a dynamic that may eventually inform decisions about treatment intensification or early transition to surgery. These observations collectively highlight post‐TNT ctDNA as the most informative timepoint for identifying minimal residual disease and stratifying recurrence risk in patients undergoing TNT.

Several non‐TNT rectal cancer studies reinforce these associations. Molinari et al. reported significantly poorer DFS among patients with persistent ctDNA after neoadjuvant therapy or surgery [28]. Wang et al. showed that combining ctDNA dynamics with MRI markedly improved prediction of pathological complete response [29]. In NEORECT, Mögele et al. demonstrated that rising ctDNA predicted metastatic progression, while Gervaso et al. observed that undetectable pre‐CRT ctDNA correlated with nodal clearance and favorable outcomes [30]. These findings support the biologic plausibility that ctDNA reflects true tumor regression even when radiologic or pathologic changes lag behind.

The patterns identified in our review fit well within the broader colorectal cancer literature. Across multiple cohorts, ctDNA clearance after multimodality therapy correlates with deeper pathologic response and improved long‐term outcomes, whereas persistent detection consistently identifies patients at risk for molecular residual disease. The prognostic significance seen in GEMCAD and NOMINATE [17, 19] echoes results from larger colorectal series, such as those by Jeanne Tie and colleagues, showing that postoperative ctDNA positivity strongly predicts recurrence [12, 31]. Collectively, these data support ctDNA as a biologically grounded, early marker of response in LARC—one that complements MRI‐based assessment and has potential to guide decisions regarding organ preservation and intensified surveillance. In this context, the combination of ctDNA with MRI tumor regression grading as explored by Alden et al. [22] who demonstrated that all patients with a complete or good radiographic response had undetectable ctDNA post‐TNT, suggests that multimodal integration may improve both sensitivity and specificity beyond either tool alone. As watch‐and‐wait strategies gain traction following TNT, accurately identifying patients suitable for non‐operative management remains a key clinical challenge. While MRI and endoscopic assessment remain central, they may not reliably detect microscopic residual disease [32]. Accordingly, ctDNA could provide complementary molecular information to help guide decisions between surgery and surveillance, particularly in patients with near‐complete clinical response.

Additional studies further illustrate the tight relationship between ctDNA kinetics and treatment response. Declines in variant allele frequencies after chemoradiotherapy and high rates of ctDNA clearance among complete responders have been described [33], while persistent detection reliably identifies patients with nodal disease or incomplete tumor regression [34]. The role of ctDNA in identifying minimal residual disease has also been demonstrated outside of TNT settings, with Liu et al. [35] showing that ctDNA detection before surgery is a strong independent predictor of recurrence. Although investigations in other gastrointestinal cancers such as pancreatic, hepatocellular, and gastric cancers extend the relevance of ctDNA [36, 37, 38], the strongest and most clinically actionable evidence remains within colorectal cancer. Real‐world data further reinforce these observations. In a multi‐institutional cohort, TNT was associated with higher rates of pathological complete response and greater use of non‐operative management compared with standard chemoradiotherapy, supporting its evolving role in organ preservation in routine clinical practice [39].

Finally, the capacity of ctDNA to provide non‐invasive molecular profiling, including detection of resistance mutations such as EGFR T790M, ESR1, or KRAS, underscores its broader relevance within precision oncology [10, 26]. While these applications extend beyond TNT‐treated LARC, they highlight the versatility of ctDNA as a platform for disease monitoring, early relapse detection, and adaptive treatment planning.

## 5 Conclusion

The available evidence indicates that ctDNA dynamics serve as a clinically meaningful molecular marker of treatment response in patients with LARC receiving TNT. While baseline ctDNA detection is near‐universal, it lacks discriminatory value for predicting outcomes. By contrast, ctDNA status at the completion of TNT or immediately prior to surgery carries the greatest predictive and prognostic significance. ctDNA clearance at these later timepoints corresponds to clinical and pathological complete response, whereas persistent or re‐emergent positivity identifies patients at elevated risk of residual disease and recurrence. These findings underscore that the timing of ctDNA assessment is as clinically meaningful as the result itself, and highlight its potential to refine risk stratification, support organ‐preservation strategies, and inform post‐treatment surveillance. However, heterogeneity in assay platforms, sampling schedules, and study designs underscores the need for standardized protocols and prospective validation before ctDNA‐guided decision‐making can be adopted into routine clinical practice.

## 6 Limitations

Our conclusions are constrained by the limited number of eligible studies, modest cohort sizes, heterogeneous TNT regimens, and variable sampling schedules. Several findings are based on exploratory or nested biomarker cohorts rather than full trial populations, introducing the potential for selection bias. Furthermore, the retrospective studies by Kim et al. and Alden et al. enrolled patients in whom ctDNA testing was ordered at clinician discretion rather than systematically, introducing additional selection bias; in particular, Alden et al.'s surgery group was enriched for poor responders, which likely underestimated ctDNA sensitivity for residual disease detection in that cohort. The lack of standardized thresholds for ctDNA positivity and variability in response definitions further limit comparability. As most studies assessed ctDNA retrospectively and did not test ctDNA‐directed clinical strategies, its prospective utility remains unproven.

## 7 Future Perspectives

Looking ahead, for clinicians managing LARC, the central question is not whether ctDNA can be detected, but whether it can meaningfully guide decisions. The emerging data suggest that post‐TNT ctDNA status may help identify patients who truly require surgery or intensified surveillance, while reassuring others who may safely pursue organ preservation. The priority is not additional exploratory biomarker analyses, but prospective trials that incorporate ctDNA into treatment algorithms and test whether acting on these signals improves outcomes. Until then, ctDNA should be viewed as a promising adjunct to, not a replacement, for careful clinical and radiologic assessment.

## Author Contributions

**Faizan Bashir:** conceptualization, methodology, writing – review and editing, supervision, investigation, writing – original draft, formal analysis. **Qurat‐ul‐Aine Bhat:** methodology, writing – review and editing; writing – original draft, investigation, formal analysis, conceptualization, supervision. **Jayden Allegakoen:** writing – review and editing, visualization. **Dorsa Shekouh:** data curation, resources. **Sajida Zaiter:** investigation, resources.

## Funding

The authors have nothing to report.

## Ethics Statement

The authors have nothing to report.

## Conflicts of Interest

The authors declare no conflicts of interest.

## Transparency Statement

The lead author Faizan Bashir affirms that this manuscript is an honest, accurate, and transparent account of the study being reported; that no important aspects of the study have been omitted and that any discrepancies from the study as planned (and, if relevant, registered) have been appropriately explained.

## Data Availability

No datasets were generated or analyzed during the current study. All information is contained within the manuscript.
